# Association between childhood family structure and longitudinal health behaviour changes in adulthood –Northern Finland birth cohort 1966 study

**DOI:** 10.1186/s12889-024-19266-3

**Published:** 2024-07-03

**Authors:** Heidi Varis, Maria Hagnäs, Ilona Mikkola, Tanja Nordström, Anja Taanila, Sirkka Keinänen-Kiukaanniemi, Eveliina Heikkala

**Affiliations:** 1 Research Unit of Population Health, University of Oulu, P.O. Box 8000, Oulu, FI-90014 Finland; 2 Wellbeing Services, County of Lapland, Rovaniemi, Finland; 3 Medical Research Center Oulu, Oulu University Hospital and University of Oulu, Oulu, Finland; 4 Arctic Biobank, Infrastructure for Population Studies, Faculty of Medicine, Northern Finland Birth Cohorts, University of Oulu, Oulu, Finland; 5 Unit of Primary Care, Oulu University Hospital, Oulu, Finland; 6 Healthcare and Social Services of Selänne, Pyhäjärvi, Finland

**Keywords:** Health behaviour, Parental separation, Single-parent, Offspring, Longitudinal

## Abstract

**Background:**

Childhood family structure is considered to play a role in person’s health and welfare. This study investigated the relationships between the longitudinal changes of adult health behaviours and childhood family structure.

**Methods:**

From Northern Finland Birth Cohort 1966 questionnaires, we collected data on childhood family structure at the age of 14 (‘two-parent family’, ‘one parent not living at home/no information on father’, and ‘father or mother deceased’), and on health behaviours (smoking, alcohol consumption and physical activity status) at the ages of 31 and 46. We used the multinomial logistic regression model to estimate the unadjusted and adjusted associations between childhood family structures and the longitudinal changes between 31 and 46 years of health behaviours (four-category variables).

**Results:**

Of the study sample (*n* = 5431; 55.5% females), 7.1% of the offspring were represented in the ‘One parent not living at home/no information on father’ subgroup, 6.3% in the ‘Father or mother deceased’ subgroup and 86.6% in the ‘Two-parent family’. ‘One parent not living at home/no information on father’ offspring were approximately twice as likely to smoke (adjusted OR 2.19, 95% CI 1.70–2.81) and heavily consume alcohol (adjusted OR 1.99, 95% CI 1.25–3.16) at both times in adulthood, relative to not smoking or not heavily consume alcohol, and compared with ‘two-parent family’ offspring. We found no statistically significant associations between childhood family structure and physical activity status changes in adulthood.

**Conclusions:**

Our findings suggest that the offspring of single-parent families in particular should be supported in early life to diminish their risk of unhealthy behaviours in adulthood.

**Supplementary Information:**

The online version contains supplementary material available at 10.1186/s12889-024-19266-3.

## Background

In recent decades, divorce rates and births outside of marriage have increased in the Western world [1, 2]. The frequency of offspring living in single-parent and different family environments is higher today than before [1, 2]. In addition, childhood family structure has been associated with offspring health and welfare not only in childhood and adolescence, but also in adulthood [3–6]. Specifically, it seems that the offspring from of single-parent families are at an increased risk of poor physical and mental health outcomes later in life [7–9].

Health behaviours are well-recognized risk factors for a number of diseases [10, 11]. They also play an important role in mental well-being [10, 12]. Parental separation during childhood has shown to associate with several of these behaviours, such as smoking, excessive alcohol use, and physical inactivity, in adulthood [13–15], which could be one possible explanation for previously reported associations between childhood family structure and later health and well-being. However, the existing literature lacks population-based follow-up studies that investigate the relationships between the longitudinal changes of adult health behaviours and childhood family structure. Further knowledge on the prognostic factors of longitudinal health behaviours, particularly those related to social factors such as childhood family structure, is important. This information can help provide support for individuals in early life in order to maintain their healthy behaviours across their lifespans.

Therefore, this longitudinal study aims to investigate the association between individuals’ family structures at the age of 14 (two-parent vs. single-parent family, with two subgroups) and longitudinal changes of health behaviours, including smoking, alcohol consumption, and physical activity, between the ages of 31 and 46. Our hypothesis was that the single-parent family subgroups would be associated with offspring’s longitudinal unhealthy behaviours in our large population-based study setting.

## Methods

### Study design and study sample

**Fig. 1:**
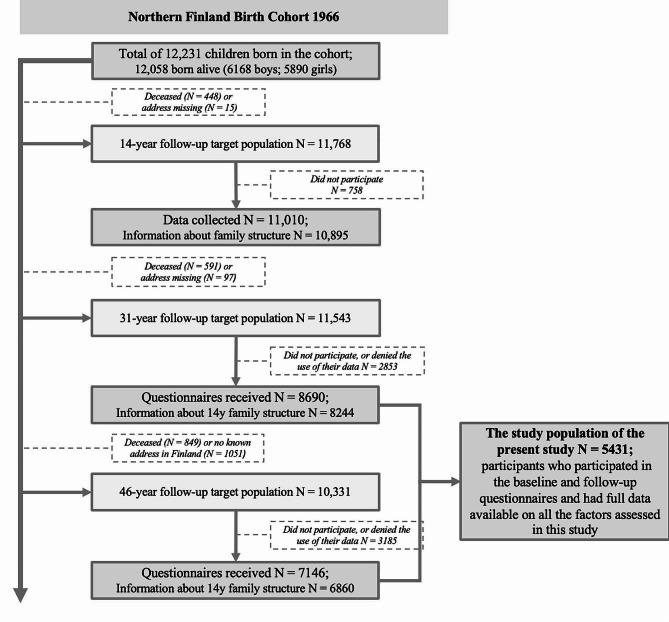
The flowchart of the study sample

This longitudinal study was based on the Northern Finland Birth Cohort 1966 (NFBC1966) project, which is a large, ongoing, prospective, general, population-based research programme in Finland’s two former northernmost provinces (Oulu and Lapland). The NFBC1966 comprises 96.3% of all live births in the regions that had expected delivery dates between 1st January 1966 and 31st December 1966 (initially a total of 12 231 individuals). The entire cohort has been followed from pregnancy (from the 24th gestational week) to subsequently predetermined timepoints (birth and the ages of 1, 14, 31 and 46). This study primarily utilized the 14-, 31- and 46-year data collection-point’s postal questionnaires, inquiring about family structure (at 14 years), health behaviours (at 31 and 46 years), and confounding factors. The 14-year data collection point was considered the baseline and the 31- and 46-year data collection points were considered the follow-ups. The study sample consisted of the 5431 participants who participated in the baseline and follow-up questionnaires and had full data available on all the factors assessed in this study. Figure 1 presents a more specific flow chart of the selection of the study sample. All the participants provided their written informed consent, and the research plan was approved by the Ethics Committee of the Northern Ostrobothnia Hospital District, Oulu, Finland. [16, 17]

### Study variables

#### Independent variable

We used family structure as the independent variable, categorized as a ‘two-parent family’ or a ‘single-parent family’, based on the information supplied by the cohort members at the age of 14. Some studies have shown that parental separation may be more detrimental e.g., to offspring’s mental health than the parental death [18, 19]. Therefore, the single-parent families were further subdivided as follows: ‘father or mother deceased’, ‘one parent not living at home (due to parental separation)’, and ‘no information on father’. The last two categories were combined due to the low number of participants in the ‘no information on father’ category (*n* = 50). A two-parent family was considered the reference. In our study sample, there were no individuals who had lost both their biological parents.

#### Outcomes

We used longitudinal health behaviours (smoking, alcohol consumption, and physical activity) between the ages of 31 and 46 years as the outcomes.

### Smoking status

We inquired about smoking by asking the following questions: ‘Have you ever smoked?’, ‘Have you ever smoked regularly, almost daily for at least a year?’, ‘Do you currently smoke?’ and ‘When was the last time you smoked?’. Based on the answers to these questions, the participants were divided into two categories at both follow-up points (31 and 46 years): (1) current/random smoker and (2) former smoker/never-smoker [20]. We formulated the following longitudinal smoking status variable from these dummy variables: (1) continued smoking (current/random smoker at 31 and 46 years), (2) started smoking (only current/random smoker at 46 years), (3) stopped smoking (only current/random smoker at 31 years), and (4) continued non-smoking (former smoker/never-smoker at 31 and 46 years). The last category was used as the reference.

### Alcohol consumption status

The participants were asked to report their consumption of alcohol beverages (yes/no). Those who answered ‘yes’ were then further asked to estimate the frequency and amount of the different alcohol beverages that they consumed. Of these estimates, we calculated the daily consumption of ethanol (EtOH) (g/day) and divided the individuals into abstainers/moderate users (< 30 g of EtOH for men and < 20 g of EtOH for women) and heavy users ( = > 30 g of EtOH for men and = > 20 g of EtOH for women) [21]. Those who were heavy users at both follow-up points were labelled ‘continued heavy using’, those who were only heavy users at 46 years, ‘started heavy using’, those who were only heavy users at 31 years, ‘stopped heavy using’, and those who were abstainers/moderate users at both timepoints, ‘continued non-heavy using’. The last category was used as the reference in the analyses.

### Physical activity status

Physical activity was estimated on the basis of the responses to the questions about the frequency and the duration of light or brisk physical activities during leisure time. Brisk activity was described as causing at least some sweating and breathlessness, and light activity was defined as causing no sweating or breathlessness. The activity frequency response options varied from once a month or less often to daily, and the response options for the duration of both light and brisk physical activities from not at all to more than 90 min. Based on the responses on the frequency, intensity and duration of leisure-time physical activity, the participants were classified into two groups: active (exercised briskly at least once a week or participated in light physical activity at least four times a week) and inactive (did not fulfil the criteria of being physically active) [22]. The longitudinal physical activity status variable was constructed as follows: (1) stayed inactive (inactive at both follow-up points), (2) decreased activity (only inactive at 46 years), (3) increased activity (only inactive at 31 years), and (4) stayed active (active at both follow-up points) (the reference).

### Confounding factors

The confounding factors were sex, highest education level until the age of 46, longitudinal self-rated health, and mother’s occupation status during pregnancy. Sex (female/male) was based on birth records. The participants were categorized on the basis of their highest education level until the age of 46 as follows: *basic education* (9 years or less), *secondary education* (10 to 12 years) and *tertiary education* (over 12 years). They were asked about their perceived health and their own estimate of their health at both follow-up points. The following longitudinal groups were formed on the basis of their reported responses to be poor or good: (1) remained poor (poor/very poor at 31 and 46 years), (2) worsened (only poor/very poor at 46 years), (3) improved (only poor/very poor at 31 years), and (4) remained good (very good/good/moderate at 31 and 46 years). Mother’s occupational status during pregnancy was divided into three categories: (1) no occupation (housewife), (2) low social class (unskilled workers, farmers and farmers’ wives), and (3) high social class (professionals and skilled workers).

### Other descriptive variables

The participants’ mother’s educational status during pregnancy was categorised as follows: low 0–4 years, intermediate 5–8 years and high ≥ 9 years.

### Statistical methods

All statistical analyses were performed using IBM SPSS Statistics, version 28 (IBM Corporation and its licensors 1989, 2021). Contingency tables were used to observe the distributions of longitudinal health behaviours and the confounding factors of the family structure categories, as well as the distribution of confounding factors in the categories of the outcomes. The statistical significance of these distributions was tested by Pearson’s Chi Square tests. We used multinomial logistic regression to evaluate the associations between family structure at 14 years and longitudinal health behaviours (smoking, alcohol consumption, and physical activity) between the ages of 31 and 46 years, using the two-parent family as the reference category. The associations were presented as unadjusted and adjusted for confounding factors, including sex, highest education level until the age of 46, longitudinal self-rated health, and mother’s occupational status during pregnancy. The odds ratios (OR) and their 95% confidence intervals (95% CI) are presented as main results. Predicted probabilities were calculated from adjusted multinomial logistic regression models separately for each outcome by using a SPSS matrix example presented elsewhere [23]. Confounding variables were considered as categorical ones in these analyses. Differences in the distribution of sex, mother’s education and mother’s occupational status between the participants and non-participants cohort members were evaluated through cross-tabulation (using Pearson’s chi-squared test) to analyse the representativeness of the study sample. *P*-values less than 0.05 were considered statistically significant. All the tests were two tailed. Pseudo-R square measure (Nagelkerke) was provided as an indicator of model fit.

## Results

Table 1 presents the characteristics of the study population, stratified by family structure at the age of 14 (*n* = 5431). The offspring belonging to the ‘one parent not living at home/no information on father’ family structure subgroup were significantly more likely to be female (63.4% vs. 55.5% [two-parent family] and 57.1% [father/mother deceased], *p* = .010), belong to the ‘continued smoking’ (31.6% vs. 19.5% [two-parent family] and 23.6% [father/mother deceased], *p* < .001), and ‘continued heavy using’ (6.0% vs 3.2% [two-parent family] and 5.5% [father/mother deceased], *p* = .003) categories, and less likely to belong to the ‘remained good’ category of self-reported health status (89.6% vs. 95.0% [two-parent family] and 93.6% [father/mother deceased], *p* < .001) than the offspring in the other family structure subgroups. A higher percentage of the offspring of the two-parent families than of the other family structure subgroups had tertiary education (28.3% vs. 27.4% [one parent not living at home/no information on father], and 20.7% [father/mother deceased], *p* = .001). The distribution of included confounders statistically significantly varied within the categories of the outcome variables (Supplement 1). There were some differences (*p* < .001) between the participants and non-participants, e.g. the participants were more likely females and had higher number of highly educated mothers and mothers with high occupational status (Supplement 2).

**Table 1:** Characteristics of the study sample, stratified by childhood family structure at the age of 14 (*n* = 5431)

	Two-parent family *n* = 4705 (86.6%)			One parent not living at home/No information on father *n* = 383 (7.1%)			Father or mother deceased *n* = 343 (6.3%)		*P*-value
	n	%		n	%		n	%	
**Sex**									
Female	2611	55.5		243	63.4		196	57.1	0.010
Male	2094	44.5		140	36.6		147	42.9	
**Longitudinal changes between 31 and 46 years**									
**Smoking status**									
Continued smoking	916	19.5		121	31.6		81	23.6	< 0.001
Started smoking	319	6.8		27	7.0		24	7.0	
Stopped smoking	641	13.6		63	16.4		59	17.2	
Continued non-smoking	2829	60.1		172	44.9		179	52.2	
**Alcohol consumption status**									
Continued heavy using	152	3.2		23	6.0		19	5.5	0.003
Started heavy using	401	8.5		35	9.1		22	6.4	
Stopped heavy using	177	3.8		22	5.7		9	2.6	
Continued non-heavy using	3975	84.5		303	79.1		293	85.4	
**Physical activity status**									
Stayed inactive	473	10.1		50	13.1		41	12.0	0.063
Decreased activity	531	11.3		56	14.6		6.8	12.5	
Increased activity	741	15.7		53	13.8		42	12.2	
Stayed active	2960	62.9		224	58.5		217	63.3	
**Self-reported health status to be poor or good**									
Remained poor	26	0.6		4	1.0		0	0	< 0.001
Worsened	127	2.7		19	5.0		18	5.2	
Improved	82	1.7		17	4.4		4	1.2	
Remained good	4470	95.0		343	89.6		321	93.6	
**Highest education level until age of 46**									
Basic or less	153	3.3		18	4.7		23	6.7	0.001
Secondary	3221	68.5		260	67.9		249	72.6	
Tertiary	1331	28.3		105	27.4		71	20.7	
**Mother’s education (during pregnancy)**									
Low 0–4 years	402	8.6		21	5.6		44	13.0	< 0.001
Intermediate 5–8 years	2570	55.1		206	54.5		205	60.7	
High ≥ 9 years	1690	36.3		151	39.9		89	26.3	
**Mother’s occupational status (during pregnancy)**									
No occupation	1376	29.2		106	27.7		114	33.2	0.418
Low social class	2720	57.8		230	60.1		192	56.0	
High social class	609	12.9		47	12.3		37	10.8	

Data are presented as numbers and percentages for the study sample. Differences between family structure categories in characteristics were identified through crosstabulation (using Pearson’s Chi Square test).

Table 2 presents the association between the offspring’s family structure subgroups at 14 years and changes in smoking behaviour between ages 31 and 46 years relative to continued non-smoking. The offspring belonging to the ‘one parent not living at home/no information on father’ family structure subgroup had over double the odds of belonging to the ‘continued smoking’ category in comparison to offspring in the ‘two-parent family’ after adjustments for sex, highest educational level until the age of 46, longitudinal self-reported health status, and mother’s occupational status during pregnancy (OR 2.19, 95% CI 1.70–2.81). In the unadjusted model, the offspring who had experienced a parental death before the age of 14, also had higher odds of belonging to the ‘continued smoking’ category (OR 1.40, 95% CI 1.06–1.84), but the association attenuated to non-significant after adjustments (OR 1.30, 95% CI 0.98–1.72). Statistically significant associations were also detected between the ‘one parent not living at home/no information on father’, ‘father/mother deceased’, and ‘stopped smoking’ subgroups (OR 1.70, 95% CI 1.25–2.30 [one parent not living at home/no information on father], and OR 1.42, 95% CI 1.04–1.94 [father/mother deceased]).

**Table 2:** Associations between family structure at 14 years and smoking status between 31 and 46 years

Family structure	Continued smoking	Started smoking	Stopped smoking	Continued non-smoking
	**Unadjusted OR (95% CI)**			
**One parent not living at home/No information on father**	**2.17** (1.70–2.77)	1.39 (0.91–2.12)	**1.62** (1.20–2.19)	Ref.
**Father or mother deceased**	**1.40** (1.06–1.84)	1.19 (0.77–1.85)	**1.50** (1.07–1.98)	Ref.
**Two-parent family**	Ref.	Ref.	Ref.	
	Adjusted OR (95% CI)*			
**One parent not living at home/No information on father**	**2.19** (1.70–2.81)	1.33 (0.87–2.03)	**1.70** (1.25–2.30)	Ref.
**Father or mother deceased**	1.30 (0.98–1.72)	1.17 (0.75–1.82)	**1.42** (1.04–1.94)	Ref.
**Two-parent family**	Ref.	Ref.	Ref.	

* Adjusted for sex, cohort members’ highest educational level until the age of 46, longitudinal self-reported health status, and mother’s occupational status during pregnancy. Statistically significant values are in bold. OR = Odds Ratio, CI = Confidence Interval

Pseudo R-Square (Nagelkerke = 0.071)

Table 3 presents the association between the offspring’s family structure subgroups at 14 years and changes in heavy use of alcohol between ages 31 and 46 years relative to continued non-heavy using. In the adjusted model, the offspring of the ‘one parent not living at home/no information on father’ subgroup had nearly double the odds of belonging to the ‘continued heavy using’ category in comparison to the offspring of the ‘two-parent family’ subgroup (OR 1.99, 95% CI 1.25–3.16). The offspring of the ‘father or mother deceased’ subgroup had over one-and-a-half-times higher odds of belonging to the ‘continued heavy using’ category than the offspring of two-parent families (OR 1.70, 95% CI 1.04–2.77), but this association did not reach statistical significance after adjustments (OR 1.64, 95% CI 0.99–2.71). There were statistically significant associations between the ‘one parent not living at home/no information on father’ and the ‘stopped heavy using’ subgroups as well (adjusted OR 1.73, 95% CI 1.09–2.76).

**Table 3:** Associations between family structure at 14 years and alcohol consumption status between 31 and 46 years

Family structure	Continued heavy using	Started heavy using	Stopped heavy using	Continued non-heavy using
	**Unadjusted OR (95% CI)**			
**One parent not living at home/No information on father**	**1.99** (1.26–3.13)	1.15 (0.80–1.65)	**1.63** (1.03–2.58)	Ref.
**Father or mother deceased**	**1.70** (1.04–2.77)	0.74 (0.48–1.16)	0.69 (0.35–1.36)	Ref.
**Two-parent family**	Ref.	Ref.	Ref.	
	Adjusted OR (95% CI)*			
**One parent not living at home/No information on father**	**1.99** (1.25–3.16)	1.19 (0.82–1.72)	**1.73** (1.09–2.76)	Ref.
**Father or mother deceased**	1.64 (0.99–2.71)	0.72 (0.46–1.12)	0.65 (0.33–1.30)	Ref.
**Two-parent family**	Ref.	Ref.	Ref.	

* Adjusted for sex, cohort members’ highest educational level until the age of 46, longitudinal self-reported health status, and mother’s occupational status during pregnancy. Statistically significant values are in bold. OR = Odds Ratio, CI = Confidence Interval

Pseudo R-Square (Nagelkerke = 0.071)

Table 4 shows the association between the offspring’s family structure subgroups at 14 years and changes in physical activity between ages 31 and 46 years relative to staying active. The ‘one parent not living at home/no information on father’ subgroup was associated with 1.40 times higher odds of belonging to the ‘stayed inactive’ category, and 1.39 times higher odds of belonging to the ‘decreased activity’ category than those in the two-parent family subgroup. However, controlling for confounding factors diluted these significant associations. Based on the adjusted predicted probabilities, the highest difference between the ‘two-parent family’ subgroup and the other subgroups was found in continued smoking (0.23 for the ‘two-parent family’ subgroup and 0.49 for the ‘one parent not living at home/no information on father’ subgroup) (Supplement 3).

**Table 4:** Associations between family structure at 14 years and physical activity status between 31 and 46 years

Family structure	Stayed inactive	Decreased activity	Increased activity	Stayed active
	**Unadjusted OR (95% CI)**			
**One parent not living at home/No information on father**	**1.40** (1.01–1.93)	**1.39** (1.03–1.89)	0.95 (0.69–1.29)	Ref.
**Father or mother deceased**	1.18 (0.84–1.67)	1.11 (0.79–1.55)	0.77 (0.55–1.09)	Ref.
**Two-parent family**	Ref.	Ref.	Ref.	
	Adjusted OR (95% CI)*			
**One parent not living at home/No information on father**	1.28 (0.92–1.79)	1.36 (1.0-1.86)	0.93 (0.68–1.27)	Ref.
**Father or mother deceased**	1.08 (0.76–1.55)	1.04 (0.74–1.47)	0.75 (0.53–1.05)	Ref.
**Two-parent family**	Ref.	Ref.	Ref.	

* Adjusted for sex, cohort members’ highest educational level until the age of 46, longitudinal self-reported health status, and mother’s occupational status during pregnancy. Statistically significant values are in bold. OR = Odds Ratio, CI = Confidence Interval

Pseudo R-Square (Nagelkerke = 0.064)

## Discussion

In the present prospective cohort study of 5431 participants, we detected an association between childhood family structure at the age of 14 and adulthood health behaviours between 31 and 46 years. The offspring of the ‘one parent not living at home/no information on father’ subgroup were two times more likely to smoke and heavily consume alcohol in adulthood, relative to not smoking or not heavily consume alcohol, and compared with the offspring of two-parent families. These longitudinal results remained significant, even after we controlled for confounding factors (sex, cohort members’ highest educational level until the age of 46, longitudinal self-reported health status, and mother’s occupational status during pregnancy). The offspring of the single-parent family subgroup had higher odds of stopping smoking and heavy use of alcohol in adulthood, but these associations were smaller in magnitude. Longitudinal physical activity status was not associated with the childhood family structure subgroups.

We found that smoking and a high consumption of alcohol in adulthood were significantly more common among the offspring of the ‘one parent not living at home/no information on father’ subgroup than among the offspring of two-parent families. This is in line with previous studies that have shown that parental separation during childhood is associated with an increased risk of smoking and excessive drinking in adolescence and adulthood [5, 24–29]. The present study, however, offers important, additional knowledge on the association between childhood family structure and the longitudinal changes of smoking and alcohol consumption in adulthood. Even though our data did not cover late adolescence and early adulthood, it is possible that offspring who have experienced parental separation or parental absence in childhood initiate their smoking and alcohol behaviour in adolescence and/or early adulthood [26, 30]. An association has been found between earlier onset of smoking and a higher risk of nicotine dependence in adulthood [31], as has a link between earlier alcohol initiation and the risk of alcohol dependence in later life [32]. Therefore, to prevent smoking and alcohol initiation, children who experience parental absence should be supported, and youth smoking and alcohol consumption should be intervened in as early as possible. We found higher odds of stopping smoking and heavy using of alcohol in adulthood to be associated with the offspring of the single-parent family subgroup (in terms of smoking in both the single-parent subgroups and alcohol consumption in the ‘one parent not living at home/no information on father’ subgroup). It is possible that smoking and heavy use of alcohol may decrease over time in certain subsets in the single-parent family subgroups.

In the current study, we observed no association between longitudinal physical activity status and the childhood family structure subgroups after taking confounders into account. Previous reports have observed that the offspring of single-parent families are less physically active in childhood and adolescence than those of two-parent families [33, 34]. A British Birth Cohort Study examined physical activity in adulthood in relation to parental separation in childhood and found an association between parental separation and physical inactivity [15]. It may be that other factors, such as socioeconomic factors, rather than childhood family structure itself, explain the physical activity of Northern Finns status in adulthood.

Parental separation has shown to have both short- and long-term consequences for several domains of the offspring’s societal functioning [7, 35]. From a speculative point of view, our findings may be related to social problems that have been reported in single-parent families, such as the loss of a parent as a role-model [7], parents spending less time with their children [35] and economic hardship [7, 35]. Low parental involvement may offer increased access to risky behavior such as alcohol consumption [36]. Economic hardships in childhood have also shown to associate with smoking in adulthood [37]. Moreover, the offspring of a divorced family may suffer from diminished emotional security, elevated psychological stress, and decreased social and psychological maturation [38], which may in turn influence risk behaviours (coping with emotional problems) including alcohol use and smoking [36, 39]. However, it is worth noting that some offspring may have a support network (e.g. grandparents and other relatives) who can fulfil the need for adequate support, such as being present for the children and fostering emotional security. Due to electronic forms of communication and grandparents’ longevity, the closeness and contact of support networks may be greater today than in previous generations. [40] Congruently, the frequency of mental health disorders seems to be higher among offspring from single-parent families than offspring from two-parent families [7, 9]. This may also act as one potential explanation for our results, as mental health disorders correlate highly with smoking and excessive use of alcohol [10, 12]. The findings of the present study, like those of previous reports, underline the impact of an individual’s childhood family structure on their welfare in adulthood. In turn, parental separation could be favourable for the offspring in some cases, because marital conflict can cause a conflictual, abusive or negligent family situation [41]. It has been indicated that marital conflict rather than family breakdown may be primarily responsible for the problems of offspring affected by parental separation [42].

The major strength of the present study is its large birth cohort study population (*n* = 5431), and its longitudinal study design with a 15-year follow-up of the relevant health behaviours during adulthood. The study participant rate also remained high despite the long follow-up, from the age of 14 to 46. Furthermore, our study population included participants from both rural and urban areas of a large part of Finland, which increases the generalizability of the present results in addition to the abovementioned elements.

However, a few limitations need to be addressed. We had insufficient data on when parental separation or death had occurred. Our data capture only biological parents, thus there is no information available on e.g., whether offspring lived with a stepparent or not. In addition, we used self-reported data, which is likely to be susceptible to recall and social desirability biases; under-reporting of unhealthy habits and over-reporting of healthy habits are well-recognised phenomenon in research based on self-reported data [43]. There may be potential bias resulting from attrition, specifically taking into account that variation in health indicators may predict survey dropout [44]. In the analysis of representativeness, there were some differences between the participants and non-participants cohort members, e.g. the participants were more likely females and had higher number of highly educated mothers and mothers with high occupational status. This should be considered in interpreting our results. It is a well-acknowledged phenomenon in longitudinal surveys that (for example) lower levels of education and a lower income predict increasing odds of dropping out [44]. There are many factors influencing family dynamics that cannot be studied in this study context. Additionally, family structures have become more heterogeneous (due to increases in never-married, single parents, divorce, cohabitation, same-sex parenting, multi-partnered fertility, and co-residence with grandparents) in recent decades [40], which may be associated with the later life health behaviour of offspring. At the same time, it should be noted that even if the distribution of the family structure categories should change over time, its exogenous relationship with the outcome may not. Family structure heterogeneity and the factors influencing family dynamics would be interesting to study in other study samples in future research.

## Conclusions

In conclusion, in this longitudinal cohort study of 5431 participants, we observed an association between living in a single-parent family in childhood and longitudinal smoking and high consumption of alcohol in adulthood at the ages of 31 to 46. The findings of the present study add to the knowledge on the possible long-term impacts of childhood family structure on individuals’ health behaviours and welfare. Active research on the associations between childhood family structure and the offspring’s later health behaviours and welfare should continue, and it should include possible protective factors, extending the perspective to the next generation. Parental loss or separation may be a challenge for offspring and all family members, and they can have long-term consequences in later health behaviours. Clinicians working in primary care clinics should recognise patients who have unhealthy lifestyle habits and promote their health. Specifically, single parents and their offspring in vulnerable situations may need more support in aiming towards healthy lifestyle habits.

### Electronic supplementary material

Below is the link to the electronic supplementary material.

Supplementary Material 1

Supplementary Material 2

Supplementary Material 3

## Data Availability

NFBC data are available from the University of Oulu, Infrastructure for Population Studies. Permission to use the data can be obtained for research purposes via an electronic material request portal. In our use of the data, we followed the EU General Data Protection Regulation (679/2016) and the Finnish Data Protection Act. The use of personal data is based on a cohort participant’s written informed consent in their most recent follow-up study, which may cause limitations to its use. Please contact the NFBC project centre (NFBCprojectcenter@oulu.fi) or visit the cohort website (www.oulu.fi/nfbc) for more information.

## Abbreviations

CI: Confidence intervals
EtOH: Ethanol
NFBC1966: Northern Finland Birth Cohort 1966
OR: Odds ratios
